# Characterizing preferences of fishermen to inform decision-making: A case study of the Pacific halibut (*Hippoglossus stenolepis*) fishery off Alaska

**DOI:** 10.1371/journal.pone.0212537

**Published:** 2019-03-01

**Authors:** Elizabeth Figus, Keith R. Criddle

**Affiliations:** Juneau Center for Fisheries and Ocean Sciences, University of Alaska Fairbanks, Juneau, Alaska, United States of America; Maurice Lamontagne Institute, CANADA

## Abstract

In fisheries management, *ex-ante* analysis of fishermen’s preferences can provide reliable insights into specific characteristics of regulatory alternatives that are desirable, objectionable, or important, in the judgement of fishermen. This knowledge could facilitate consideration by fishery managers of additional regulatory alternatives with high likelihoods of meeting program objectives, minimal disruption to fishing operations and lifestyles, and high levels of acceptance and compliance from the fishing fleet. In this case study, we interviewed Pacific halibut fishermen (n = 76) in four communities across Southeast Alaska, to document their preferences about different types of data collection methods on their vessels. We demonstrate how to use interviewing to gather preference data from a relatively small group of fishermen and get a reliable snapshot of preferences across an entire region. Pairwise comparisons from interviews were analyzed using a three-stage analytic hierarchy process model. Results characterize the variability of fishermen’s preferences about data collection methods.

## Introduction

Determining the sustainable use of fisheries resources involves balancing multiple social objectives [1] and evolving social objectives stimulate changes in fisheries policies [2]. Different stakeholder groups (e.g., fishermen, scientists) often hold different preferences which can be characterized as different weights on the various elements of society’s multiple objectives [3]. Understanding these preferences and how they relate to trade-offs between ecosystem services can enhance planning [4,5]. Although it may be unrealistic to expect that solutions to a given management challenge will be preferred by all interest groups [4], achieving consensus across multiple stakeholder groups is possible [6].

Fishermen often have expert knowledge and understanding of their local regions and communities that outside experts do not possess [7,8]. Documenting preferences of stakeholders—including fishermen—in structured, systematic ways may improve marine spatial planning [9]; provide information about risks and trade-offs for maximizing resilience in the face of climate change [6]; provide guidance about local conditions or vulnerabilities to enhance local adaptation to environmental changes [10]; help define social objectives in fisheries management [11–13]; assist fisheries managers in predicting impacts of harvest strategies [14]; and, serve as a basis for evaluating investment or diversification strategies in the fishing industry [15,16]. Increased stakeholder participation in the regulatory process can also increase compliance with fishery regulations [17].

For these reasons, there has long been a call to increase stakeholder participation in environmental regulatory processes beyond public comments [18,19] to include structured, systematic information gathering. Academics have refined methods for documenting stakeholder preferences for incorporation into the design of management strategies [20–25]. However, it remains uncommon for fisheries managers to analyze fishermen’s preferences for incorporation in the decision-making process in structured or systematic ways.

Analyzing and incorporating preferences of fishermen may be uncommon because agency personnel are unfamiliar with social science methods [26], because the human dimensions objectives of a management strategy are not clearly articulated [25], or because data collection methods such as the public comment process may assign disproportionate weight to input from particular stakeholders [23]. Issues such as unfamiliarity with social science methods might only be resolved through capacity building within management institutions (e.g., hiring new staff). Guarding against assigning disproportionate weight to input from particular stakeholders could be addressed by refining the use of social science methods in existing processes. This research explores a mixed method of interviews and decision analysis for refining the use of social science methods in existing fisheries management processes.

The goal of this paper is to present results of a case study in Alaska demonstrating how preferences of fishermen can be documented in a structured, systematic way for incorporation into existing fisheries management processes. We assessed preferences fishermen have about four alternatives that could be considered for collecting data about commercial fishing catch and discards. We assessed preferences in three stages: (Stage 1) Determine the strength of fishermen’s ranked preferences across four hypothetical types of data collection on their vessels (called “alternatives”); (Stage 2) Determine the strength of fishermen’s preferences between pairwise comparisons of the four data collection alternatives, while considering attributes of fishing operations that might underlie those preferences; and, (Stage 3) Determine how well each alternative performs in relation to a suite of standards (called “criteria”) that influenced fishermen’s preferences. We present a case study of the Pacific halibut (*Hippoglossus stenolepis*), hereafter “halibut,” commercial fishery off Southeast Alaska. We used semistructured interviews [27] to gather the information necessary to understand the strength of preferences halibut fishermen have about data collection on their vessels, as well as criteria affecting those preferences.

### Case study

**Fig 1:**
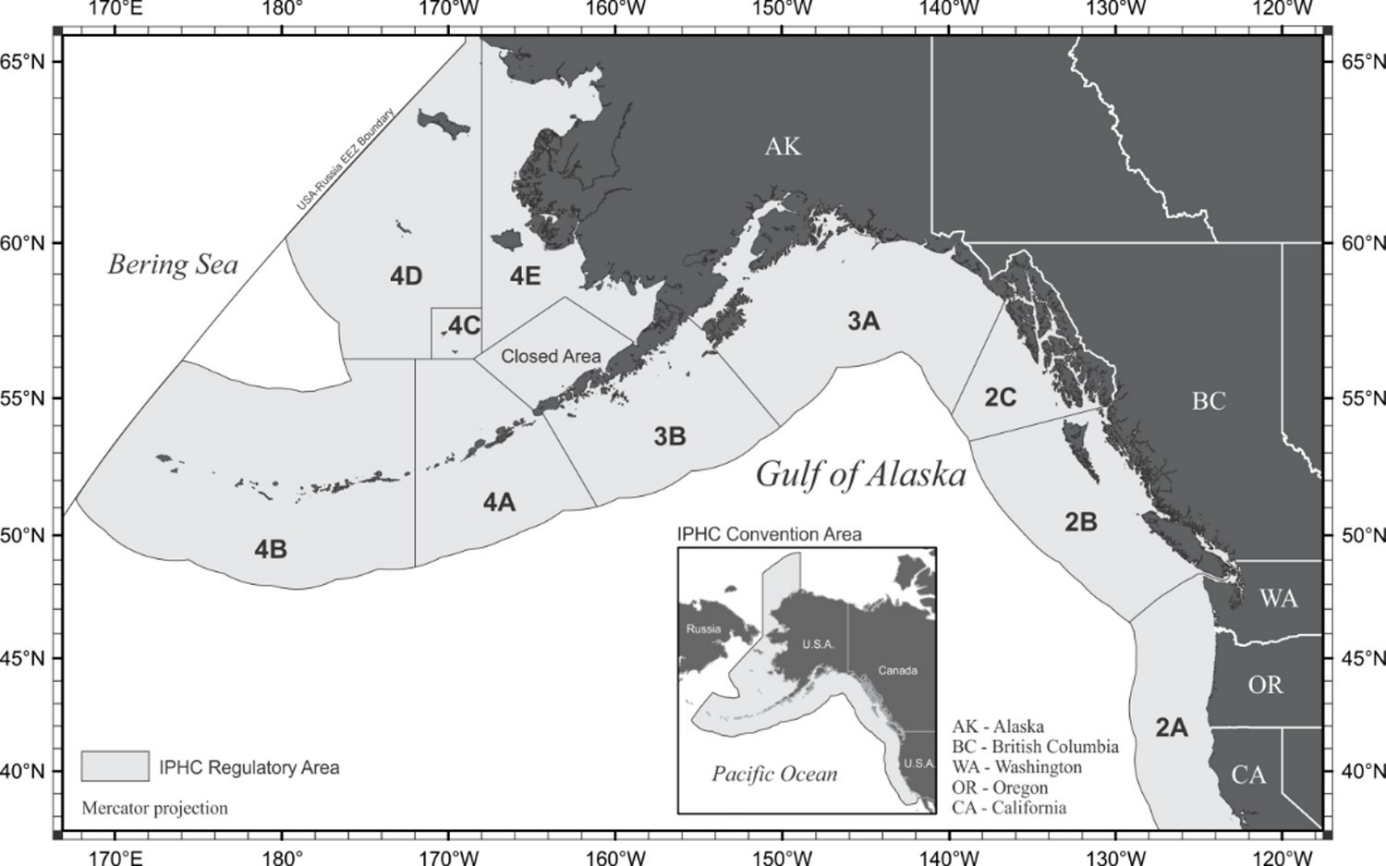
IPHC regulatory areas (courtesy of IPHC).

The Alaska region commercial fishery for halibut involves over 1,000 vessels, most of which are less than 18 m. in length overall [28]. These vessels deploy demersal longline gear—several hundred baited hooks spaced at regular intervals along a weighted line anchored to the seafloor. Longlines are retrieved after “soaking” for several hours. The halibut fishery is managed across ten regulatory areas (Fig 1) set by the International Pacific Halibut Commission (IPHC). For this study, we focused on IPHC regulatory Area 2C, which covers the Southeast Alaska region. Focusing our study in one region allowed us to analyze preferences while controlling for factors that vary across the regulatory areas (e.g., incidental catch/discard regulations). Southeast Alaska includes 28 different communities that landed a combined 100,250 tonnes of fish and shellfish in 2016, with an ex-vessel value of $218 million [29].

The halibut fishery off Alaska is managed under a system of Individual Fishing Quotas (hereafter “quota”) with mandatory dockside monitoring of all landings and very low incidence of illegal or unreported catch [30]. In 2013, the North Pacific Fishery Management Council (hereafter “Council”) extended their observer program to include vessels commercially fishing for halibut off Alaska [31]. The observer program aims to collect data on catch, bycatch, and discards of fish, interactions with protected species, and biological samples to inform the conservation and management of federal groundfish fisheries [31]. The program initially applied to vessels > 18.3 m. length overall. In 2013, the observer program was expanded to include halibut fishing vessels over 12.2 m. in length. Since 2013, fishing vessels over 12.2 m. long must register to be randomly selected to carry an observer. The program continues to evolve, and at the time of this publication a halibut vessel selected to take an observer carries the observer for the duration of a single fishing trip. The observer records characteristics of fishing sets while keeping a tally of the different species that come up on each hook of the longline gear.

In the halibut fishery, the observer program is intended to systematically collect data about undersized halibut and other fish species discarded at sea by halibut fishermen. However, expansion of the observer program to small vessels created logistical and operational challenges and was costly to the overall program (over $1,000 per day of fishing observed in 2015) [32]. For example, the Alaska Longline Fishermen’s Association (ALFA) explained:

… our members support at-sea monitoring and are willing to pay a fair share of at-sea monitoring costs … however, small boats represent 90% of the vessels directly regulated under the new observer program, and placing human observers on these vessels presents special problems [33].

During the first years of the restructured observer program, exemptions were granted to vessels unable to accommodate observers for reasons related to limited bunk space and insufficient life raft capacity [34]. The large number of these exemptions resulted in data that were not spatially representative across the halibut fleet [32,34], and led to changes in sampling design (e.g., re-organizing deployment categories, such that vessels targeting halibut were subject to carrying observers on a trip-by-trip basis, instead of for months at a time). In addition, the Council implemented an electronic monitoring [8] alternative data collection method for the halibut fleet in 2018 [35].

At the beginning of this study, we conjectured that systematic *ex-ante* (forecast) analysis of halibut fishermen’s preferences might have provided managers with reliable insights into characteristics of data collection alternatives that were desirable, objectionable, or important to fishermen. This knowledge might have facilitated serious consideration of additional data collection alternatives for halibut vessels, including EM, during the 2013 program restructure. In turn, considering EM earlier on might have mitigated exemptions in the first years of the restructured observer program, resulting in improved, spatially representative data.

## Materials and methods

**Fig 2:**
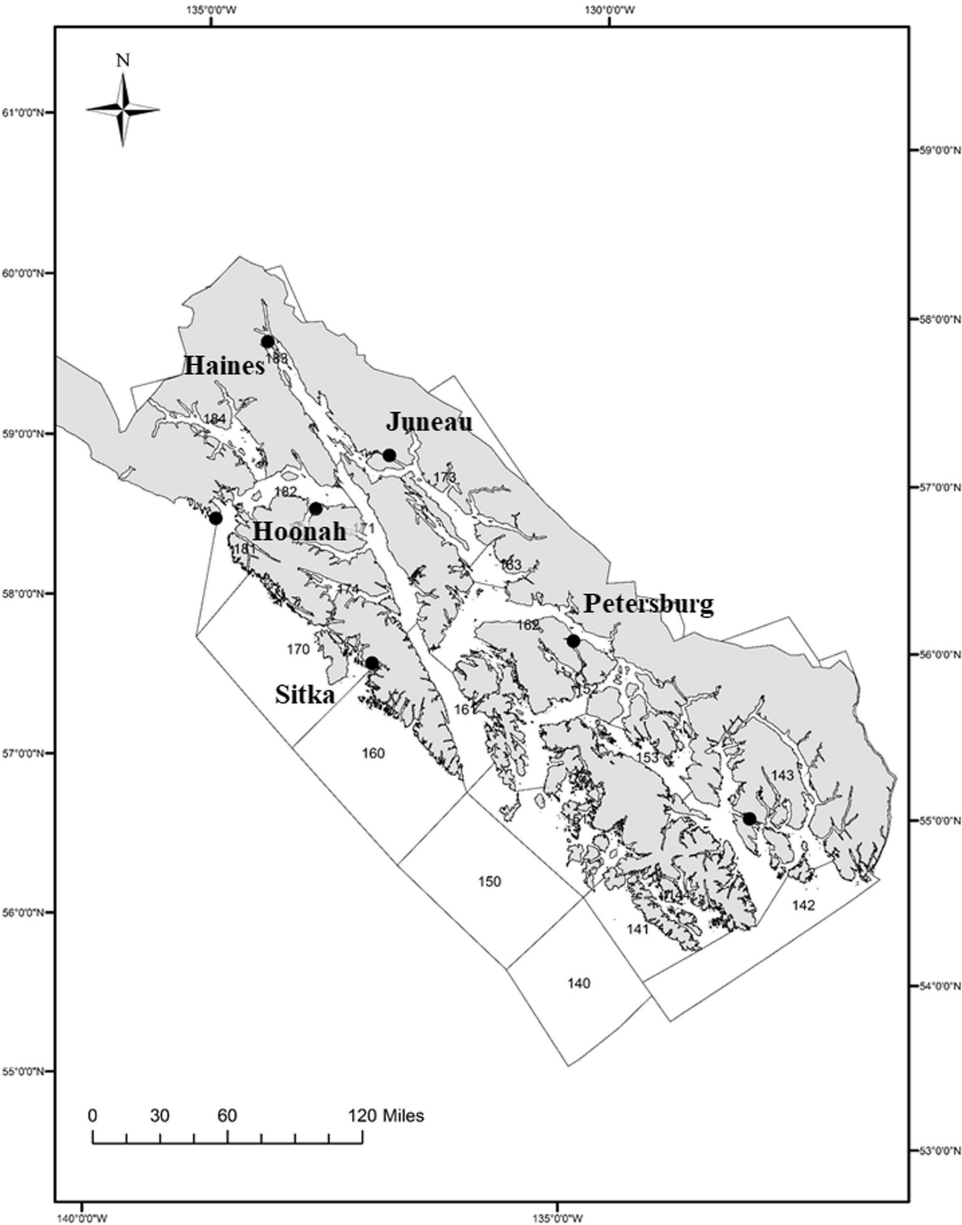
Map of regulatory area 2C, Southeast Alaska, with study communities labeled (created by the corresponding author).

We interviewed halibut fishermen in four communities across Southeast Alaska, to document their preferences about data collection (Fig 2). Three of the communities—Juneau, Petersburg, and Sitka—are the ports with the largest offloads of halibut in the region [28]. The fourth community—Hoonah—was chosen as an example of many small rural coastal communities in the region, with a strong historical connection to the halibut fishery, but with low participation in the commercial fishery today. During the winter of 2014, we sent a letter to every halibut quota holder in the four study communities (*n* = 503, S1 File) informing them of our project and inviting them to participate in an interview. Over 100 quota holders responded to those letters. We then set up semistructured interviews [27] with respondents who were available to meet during the spring 2015 and used snowball sampling to recruit additional fishermen from the study communities to participate in interviews (S2 File). The vast majority of interviews were organized through the initial letter contacts, with fewer than ten resulting solely from the recommendations of interview participants (i.e., snowball sampling). A few interview participants agreed to interviews after the initial letter and were also recommended by other fishermen to participate.

During January 2015, the interview protocol was tested with 12 halibut fishermen in the community of Haines (Fig 2). Between February and May 2015, we recorded one-on-one interviews with 76 halibut fishermen residing in Juneau, Petersburg, Sitka, and Hoonah (Fig 2 and Table A in S3 File). Interview questions were part of an interview that explored additional aspects of the halibut fishery [36]. Prior to completing interviews, the project was explained to each participant. Each individual was given a written consent form to review (S2 File). Once any questions were resolved, an individual who agreed to participate was asked to sign the consent form. At this point, we retrieved the form for our records and provided the participant with an additional un-signed form for their records.

Interview sampling was neither random nor stratified for demographic characteristics (although information about age, years of fishing experience, gender, ethnicity, and fisheries income was collected). Because demographic information about quota holders is not collected by the agencies and because we did not collect demographic information about quota holders we did not interview, we are unable to determine if the demographic characteristics of quota holders we interviewed are representative of the demographics of the population of quota holders. Because we have access to information about the type and amount of quota held by all quota holders, we were able to determine that the mix of quota types held by the interviewees was similar to the mix of quota types held in their communities. Based on information provided by the interviewees, we were able to test for differences among subsamples of our interviewees stratified by vessel size class, fishing operation characteristics, and the age and years of experience of quota holders.

There were not enough responses from underrepresented and marginalized groups (including indigenous people) in the interview group to be able to explore subgroup differences beyond age and years of fishing experience in any quantitatively or qualitatively meaningful way. There was, however, ample representation across vessel size categories and fishing operation characteristics to allow for testing of differences in preferences across these variables (S2 File). After the fact, the interview group was found to comprise an average of 20% of the quota shares held by residents across all the study communities (see Table B and Figure A in S3 File). Delineations of quota types—class A, B, C, or D—for the interview group were also reflective of the communities as a whole (see Figure A in S3 File). We did not determine representativeness of our results beyond Area 2C halibut IFQ holders.

During interviews, fishermen described characteristics of their fishing operations (Table A and Table B and Table C in S3 and S4 Files), as well as their preferences about data collection methods on their halibut fishing vessels. Interviewees were asked to carry out basic ranking as well as pairwise comparisons (S5 File) across four hypothetical alternative methods for data collection. The four hypothetical alternatives were developed at the onset of the research to reflect data collection methods considered possible but not necessarily carried through to the final analysis for 2013 expansion of the observer program. The hypothetical alternatives were intentionally general, to elicit clear responses from fishermen interviewed for this project. Hypothetical alternatives included:

1. Human Observers, i.e., agency-contracted observers to record everything that comes up on a random sample of hooks;
2. Electronic Monitoring, i.e., using agency-specified electronic cameras to record everything that comes up on every hook;
3. Detailed Logbooks (submitted to the management agency), i.e., a system where the fisherman would be responsible for recording everything that comes up on every hook (Note: vessels in this fishery are subject to random boarding at sea by U.S. Coast Guard or NOAA Enforcement officers. During the inspections, among other things, the boarding officers review permits, logbook entries, and retained catch); and,
4. Status Quo ante, i.e., maintenance of basic logbook records (submitted to the management agency) as required before the 2013 restructuring of the observer program.

While ranking and comparing the four alternatives, interviewees were encouraged to explain the criteria that influenced their decisions. Fishermen were also asked about their general thoughts concerning the observer program, previous experiences with observers, and data collection (S2 File). All interviewees completed the rank and compare exercises. We hypothesized that preferences about data collection methods might be related to characteristics of fishermen and their fishing operations. Therefore, we also gathered information on each interviewee’s age and experience, as well as their principal fishing grounds (S4 File), their community, vessel length, gear type, seasons fished, and whether they also target sablefish (*Anoplopoma fimbria*, Table A in S3 File).

The analytic approach applied in this paper is the Analytic Hierarchy Process (AHP) [37–39], a multiple criteria decision analysis approach to structured decision-making. Decision analysis methods have traditionally treated social preferences in terms of an overarching objective such as the maximization of expected monetary benefits. This has been recognized as an oversimplification of how individuals or groups might go about making decisions in fisheries management to achieve socially preferred outcomes [40,41]. However, multiple criteria decision analysis can reflect a plurality of objectives, not all of which are monetized [42].

AHP is a broadly used decision tool [43] and has long been recognized as useful in the context of fisheries management [44–47], as it solves for policy alternatives that are likely to receive the most support and the least opposition within or across stakeholder groups. AHP focuses on the preferred solution as it may be characterized by stakeholders, without being constrained to assumptions about rational-actor theory or statistical representativeness. AHP was designed for implementation in a series of face-to-face meetings [48] but can also be carried out using a serial survey/questionnaire approach [23,45,49]. Instead of meeting or communicating multiple times with groups of fishermen to gather information about their preferences, we gathered all the information needed for parameterizing the AHP model using one semistructured interview with each fisherman. A key advantage of one-on-one interviews is that each participant could spend as much time as they wanted to answer open-ended questions about the criteria influencing their preferences about data collection on their vessel.

We explored the criteria that influenced the preferences fishermen had, to determine how well each alternative performed in relation to those criteria. We defined the suite of criteria through inductive content text analysis [27], carried out in the Atlas.ti software program [50,51]. During text analysis, recurrent themes were grouped and counted to weight each one relative to the number of times it was mentioned. The following quote is one example of a response to the open-ended question relating to how the observer program has affected an interviewee’s outlook on the future of the halibut fishery:

I’ve seen a lot of changes in a lot of regulations, and—you know, and I’ve rolled with them, and I’ve just reached the point where if now I have to sleep on the floor to go fishing [to accommodate an observer], I just—I just won’t do it.

This quote was identified during text analysis in a code group titled, “Space.” In other words, the space an extra person takes up on a fishing vessel played a role in how this fisherman felt about carrying human observers (vessels that carry an observer are required to provide workspace on deck and accommodations, including a bunk and bathroom, that ensure privacy in keeping with gender identity).

During inductive coding, interviews were coded into six code groups and 58 total codes. Codes for each alternative were cross-referenced with 33 codes related to preference criteria (S8 File). The list of 33 codes corresponded to 16 criteria *themes* and were assigned positive, negative, or neutral values (S8 File). For example, if an interviewee cited space as an important consideration when thinking about an alternative but did not accompany that statement with a directional qualifier, that portion of their transcript was coded as ‘Space.’ If an interviewee cited an alternative as taking up too much space, that portion of their transcript was coded as ‘Space -,’ in relation to the given alternative. The same quote from above provides an example of the code for ‘Space’ with a negative qualifier for the human observer alternative:

Q: Is there any other way that this observer program has affected your outlook on the future of the halibut fishery?

A: I’ve seen a lot of changes in a lot of regulations, and—you know, and I’ve rolled with them, and I’ve just reached the point where if now I have to sleep on the floor to go fishing [to accommodate an observer], I just—I just won’t do it.

If an interviewee cited an alternative as being a space-saver, that portion of their transcript was coded as, ‘Space +,’ and so on. Once all the open-ended questions were coded, text analysis results were used to expand simple pairwise comparisons into a multilevel AHP model that considers weighted criteria. Finally, weighted criteria for preferences about the alternatives were added into the AHP model.

**Fig 3:**
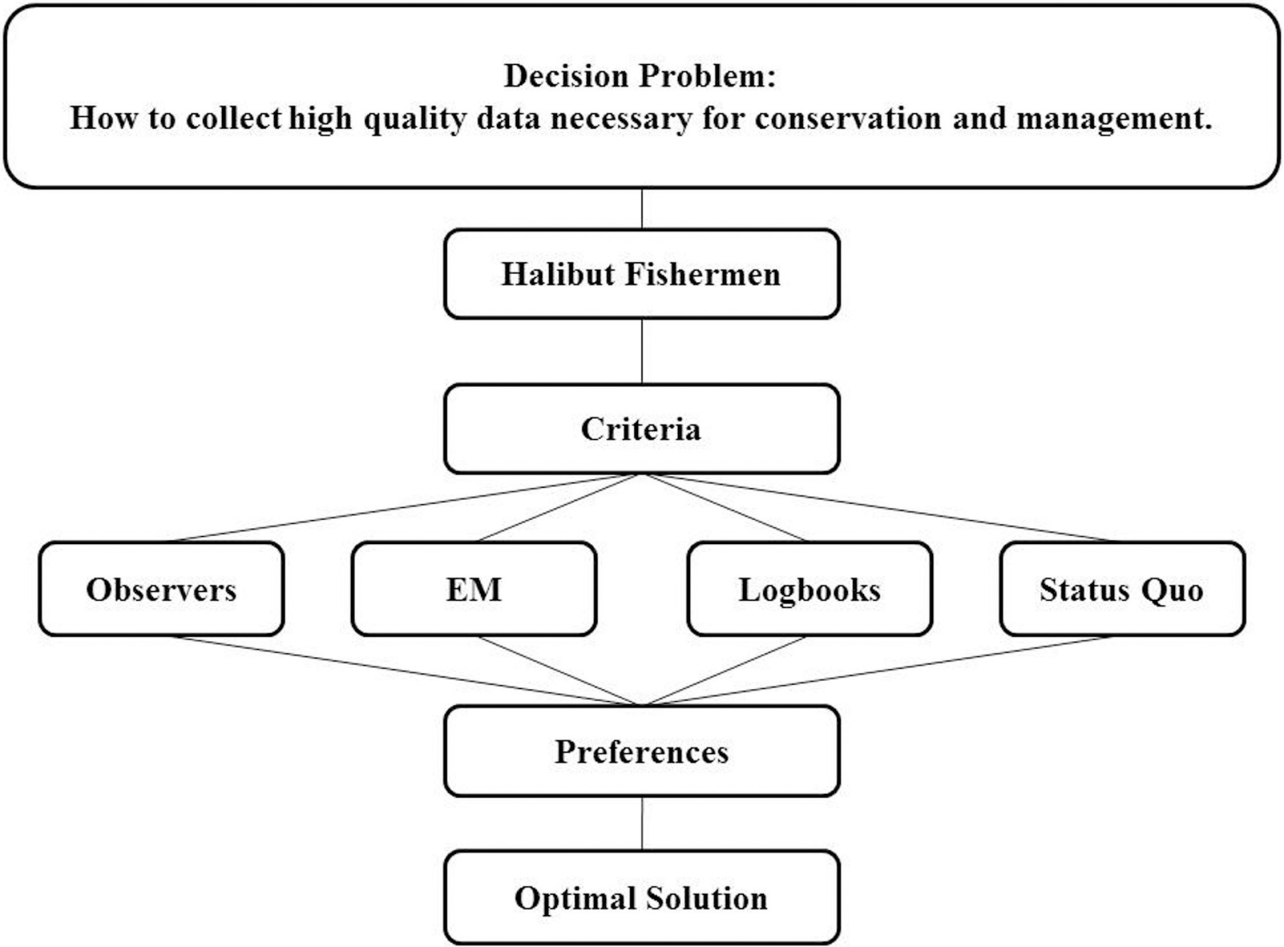
Schematic representation of the decision problem addressed in this research, with the overall goal at the top, followed by comparison criteria as they related to four data collection alternatives (observers, EM, logbooks, and the status quo), and preferences, resulting in a preferred solution (based on [38]).

Our AHP model organizes the decision problem into a hierarchy under the overall decision problem (“How to collect high quality data necessary for conservation and management”), followed by criteria fishermen consider important in judging the performance of the four data collection alternatives, and fishermen’s preferences regarding those alternatives. Our AHP uses a series of layered calculations to produce a single score for each alternative. The alternative with the highest score is assumed to be the preferred alternative by the group of fishermen interviewed for this study. The fishermen’s preferred solution reflects their judgments about what is important and their preferences about the alternatives (Fig 3).

To calculate scores for each alternative, we first assessed the median and mean central tendencies of responses, as well as the spread of responses across the interview group, to determine the strength of fishermen’s ranked preferences across four data collection alternatives. Then, we analyzed pairwise comparisons between alternatives using the AHP. Pairwise comparisons had a possible ranking across seven categories, ranging from very strong negative (1/7) to very strong positive (7, Table 1). Pairwise comparisons were then mapped into numerical values (weights), which were used to calculate a score for each alternative (S5 File) [37].

**Table 1:** Pairwise comparison ranking approach.

Importance of difference	Value assigned
Very strong positive	7
Strong positive	5
Moderate positive	3
No difference	1
Moderate negative	1/3
Strong negative	1/5
Very strong negative	1/7

To map numerical weights for our AHP, we summed the values from pairwise comparisons of each data collection alternative in each column of the matrix of pairwise comparisons. We then divided each alternative by its column total to generate a normalized pairwise matrix. Next, we divided the sum of the normalized column of the matrix by the number of alternatives (*n* = 4 in this example) to generate a weighted matrix. We multiplied the pairwise matrix by the weighted matrix, to produce a consistency vector, *λ*_max_. We calculated a consistency index (*CI*) from these scores to measure the extent to which the decisions are internally consistent in line with the Independence Axiom [52] (Eq 1). We then produced a consistency ratio (*C*_*r*_; Eq 2) from the ratio of the *CI* to a random index (*RI*) acceptable at values ≤ 10 for our example (*RI* = 0.9 for *n* = 4 alternatives) [38]. The *RI* for this research is a theoretical matrix where the alternatives have been assigned random rankings/comparison values and therefore is expected to be highly inconsistent [38]:

$$CI=\frac{\lambda_{\max}-n}{n-1}$$ (1)

$$C_{r}=\frac{CI}{RI}$$ (2)

We hypothesized that outcomes from the pairwise comparisons across alternatives might depend on characteristics of fishing operations. Therefore, we examined the sensitivity of our findings across fishing characteristics. We split the interview group into subgroups to explore how preferences might shift across attributes of fishing operations when measured using pairwise comparisons (S5 File). Subgroups included the seven characteristics that were recorded during interviews: age; experience; fishing grounds (S4 File); study community; vessel length; gear type; seasons fished; and, whether the fishermen also target sablefish (Table A in S3 File).

The AHP model results identified the strength of preferences for the four data collection alternatives held by halibut fishermen interviewed for this research. In addition, more importantly, the weighted criteria included in the AHP can be used to explore the underlying basis for those preferences.

## Results and discussion

### Stage 1: Ranking alternatives

**Fig 4:**
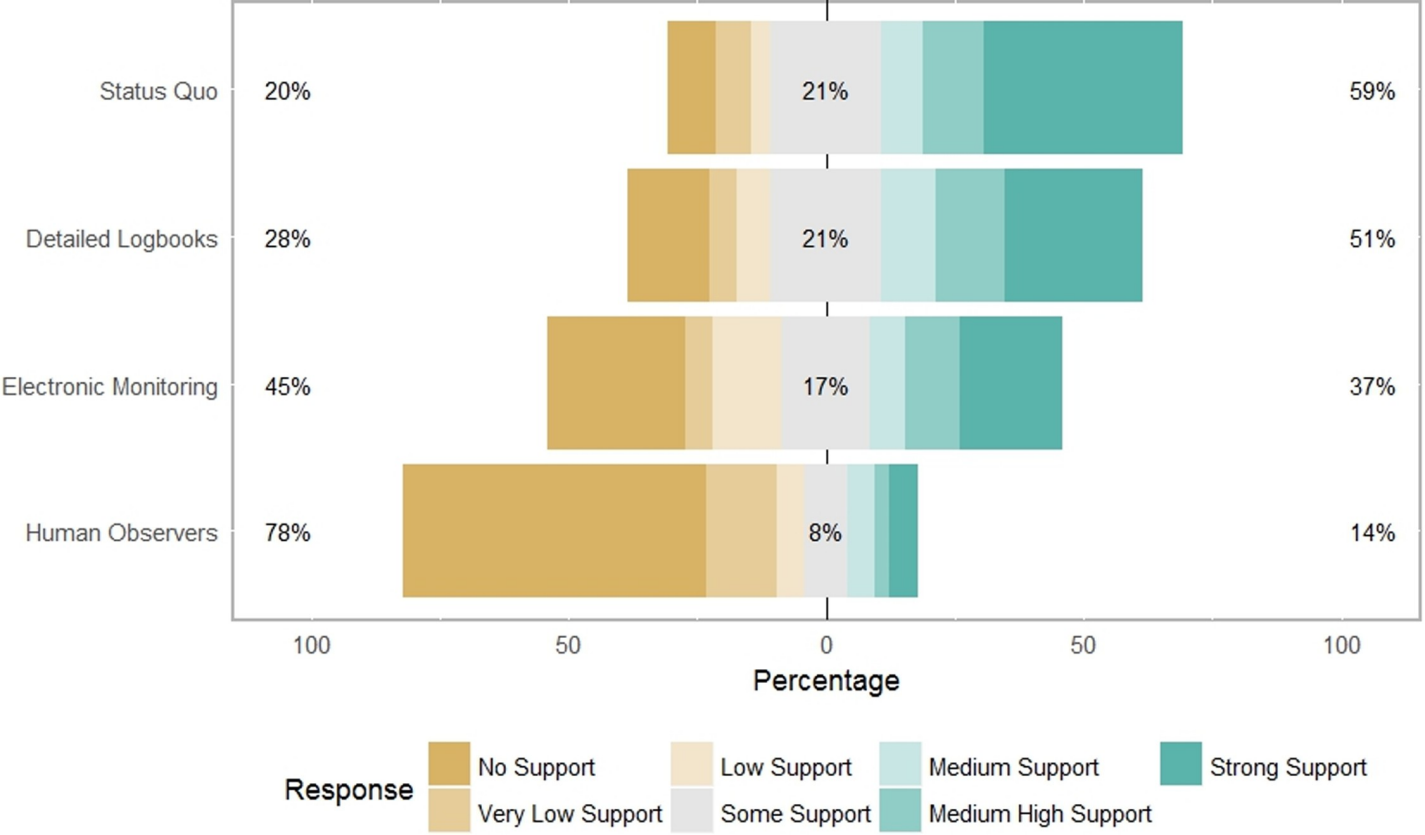
Likert analysis of rankings data across human observers, electronic monitoring, detailed logbooks, and the status quo alternatives for data collection at sea, from semistructured interviews (*n* = 75). This graphic was created using the ‘likert’ package in the R software program [53,54]. Note not all percentages add to 100, due to rounding.

Rank outputs tended to favor the status quo option, as well as keeping a detailed logbook. This suggests that people were mostly interested in self-reporting their catches. Graphical Likert analysis (Fig 4) highlighted diversity in responses and showed that a vast majority of interview participants exhibited ‘no support’ for carrying human observers on their own vessel. Only 22% of interviewees had at least ‘some support’ for the human observer alternative. In contrast, over 50% of interviewees had at least ‘some support’ for the other alternatives (status quo: 80%; detailed logbooks: 72%; and, electronic monitoring: 54%).

For each alternative, at least one interviewee assigned ‘no support’ or ‘strong support’ (see Minimum and Maximum values in Table 2). However, mean and median rank values (Table 2) both show that interviewees had strong preferences towards the status quo alternative and away from carrying human observers. Furthermore, mean and median values for the interview groups as a whole both show ‘low support’ or ‘no support’ for human observers, while the other three alternatives received at least ‘some support’ (status quo: 5–6 out of 7, detailed logbooks: 4.5–5 out of 7, and electronic monitoring: 3.8–4 out of 7).

**Table 2:** Summary of basic rankings across four alternatives for data collection (n = 75) across human observers (‘Observers’), electronic monitoring (‘EM’), detailed logbooks (‘Logbooks’), and the status quo (‘Status Quo’) alternatives for data collection at sea.

	Observers	EM	Logbooks	Status Quo
Minimum	1.0	1.00	1.00	1.00
1^st^ Quartile	1.0	1.00	3.00	4.00
Median	1.0 (no support)	4.00 (some support)	5.00 (medium support)	6.00 (high support)
Mean	2.2	3.84	4.52	5.03
3^rd^ Quartile	3.0	6.00	7.00	7.00
Maximum	7.0	7.00	7.00	7.00

This result not surprising. Fishermen can be expected to prefer alternatives that are the least costly and least disruptive. Because simple ranked alternatives provide no indication of what features drive fishermen’s preferences, we used pairwise comparisons to explore the basis for those preferences, knowledge that might be used by fisheries managers to amend alternatives in ways that improve acceptance without sacrificing efficacy.

### Stage 2: Pairwise comparisons

Analysis of pairwise comparisons produced straightforward results that were remarkably consistent. Results of simple pairwise comparisons produced the highest amount of support for status quo, followed by detailed logbooks, then electronic monitoring, and finally human observers (S5 File and Table 3). The consistency ratio for pairwise comparisons across the four alternatives was 1%, indicating remarkably consistent preferences; Saaty (1990) explains that if the value of the consistency ratio is smaller or equal to 10%, the inconsistency is acceptable. This result may be due, in part, to a tendency towards consistent and extreme preferences across the pairwise comparisons (e.g., interviewees commonly chose a value near the end of either end of the comparison spectrum). These results implied strong support throughout the interview group for the alternative that was perceived to produce the least amount of change or disruption in the lives of halibut fishermen.

**Table 3:** AHP using the geometric mean, across pairwise comparisons of human observers (‘Observers’), electronic monitoring (‘EM’), detailed logbooks (‘Logbooks’), and the status quo (‘Status Quo’) alternatives for data collection at sea.

	Observers	EM	Logbooks	Status Quo	Scores	Consistency Measure
Observers	0.08	0.06	0.08	0.10	0.08	4.01
EM	0.24	0.18	0.19	0.16	0.19	4.03
Logbooks	0.33	0.31	0.33	0.33	0.32	4.03
Status Quo	0.34	0.45	0.41	0.41	0.40	4.05
Scores	0.25	0.25	0.25	0.25	1.00	
Consistency Ratio						0.01

At the same time, a subgroup of fisherman in the interview group indicated strong support for human observers. Fishermen who ranked observers highly explained their preferences in many ways:

When we're fishing halibut we have more room because we don't have the racks in for the black cod [sablefish] gear. So we have room then for them [observers] to do their samples and we can, you know, accommodate them a hell of a lot easier. (Interviewee who indicated strong support for observers.)

… one observer trip … It was great. He bonded well with the crew, wasn’t in the way at all. Good personality. Didn’t—yeah, didn’t get in the way. (Interviewee who indicated strong support for observers.)

I sort of like having someone keeping track of what’s going on out there, a scientist who can like keep track of the health of the whole environment, because it all affects each other. (Interviewee who indicated strong support for observers.)

We wondered if the stronger acceptance of human observers among some interviewees might be correlated with categorical differences among fishing operations. Therefore, we tested the sensitivity of results from pairwise comparisons in the full interview group across subgroups of interviews clustered on demographic and fishing characteristics. Seven demographic and fishing characteristics were assessed across subgroups, including: season(s) fished out of 3 possible seasons; gear type used; whether or not they combo fish; community of residence out of 4 possible communities; under/over median age, vessel length, or years of experience; and, vessel length cutoffs at 12.2 m., 16.8 m., and 18.3 m. (Table A in S3, S4 and S5 Files). There were no evident differences among these subgroups except that interviewees who fished on vessels over 16.8 m. in length had more than twice the support for human observers (Table 4).

**Table 4:** Weight of preference among full interview group compared with subgroup of interviewees fishing on vessels greater than 16.8 m. in length, across human observers (‘Observers’), electronic monitoring (‘EM’), detailed logbooks (‘Logbooks’), and the status quo (‘Status Quo’) alternatives for data collection at sea.

	Full Group (*n* = 75)	Vessels > 16.8 m. (*n* = 13)
Observers	0.08	0.17
EM	0.19	0.23
Logbooks	0.32	0.28
Status Quo	0.40	0.32
Consistency Ratio	0.01	0.00

We wondered if the stronger acceptance of human observers among interviewees with longer vessels might be correlated with their prior experience with onboard observers in other fisheries. However, ANOVA and chi-square tests comparing preferences partitioned to reflect differences in vessel length and previous experience with human observers indicated that the observed differences were not statistically significant (S6 File, Table A and Table B in S7 File). This suggested that vessel size may merely reflect a lower opportunity cost of space needed to accommodate an observer (a non-contributing extra person) onboard the larger vessels. Eleven interviewees preferred human observers to electronic monitoring, six preferred human observers to detailed logbooks, and eight preferred human observers to the status quo. However, those interviewees that indicated a preference for human observers fished their quota on a mix of vessel lengths.

The three pairwise comparisons of data collection alternatives that included human observers had higher coefficients of variation than the three other pairwise comparisons (S5 File and Table 5). Higher coefficients of variation indicated greater levels of disagreement among interview participants about carrying human observers, as compared with preferences about the three other data collection methods (a coefficient of variation equal to zero would indicate complete consensus among interviewees).

**Table 5:** Coefficient of variation across six pairwise comparisons of human observers (‘Obs.’), electronic monitoring (‘EM’), detailed logbooks (‘Log’), and the status quo (‘SQ’) alternatives for data collection at sea.

Pairwise Comparison	Obs.- EM	Obs.- Log	Obs.- SQ	EM- Log	EM- SQ	Log- SQ
Coefficient of Variation	1.85	2.21	2.15	1.42	1.66	1.14

Nevertheless, the order of preferences for data collection alternatives was consistent and clear across the interview group as a whole.

### Stage 3: Criteria

While interesting, results from basic ranking and pairwise comparisons do not shed light on how the four data collection alternatives compare in relation to specific criteria. For example, project participants clearly preferred the status quo alternative overall, but how might they feel if they were asked to choose the most accurate data collection strategy or the least intrusive one? Questions like the one posed above can be answered by incorporating an understanding of what criteria affect preferences fishermen have about each data collection alternative.

### AHP solution

**Fig 5:**
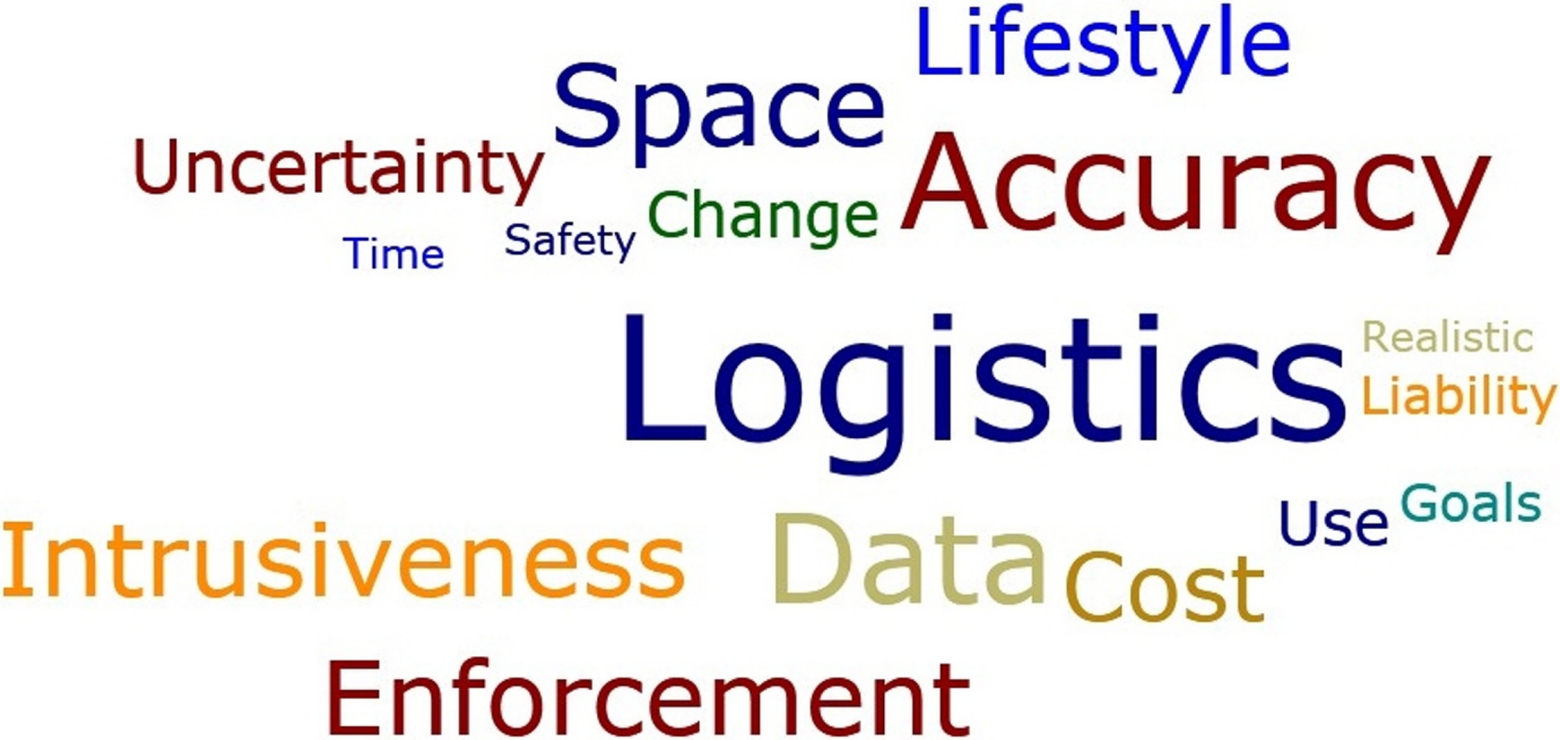
A word cloud created in the Atlas.ti program presents 16 unidirectional criteria themes arranged by size, according to their weight—how often each one was mentioned during interviews. Note that color has no intended significance in this figure.

We condensed the 33 multidirectional codes (S8 File) into the 16 unidirectional positive criteria listed in Table 6. Condensing the multidirectional criteria into unidirectional criteria facilitated weighted comparisons across criteria. Each criterion was assigned a weight corresponding to how often it was mentioned during interviews (see the ‘Total’ column in S8 File and Fig 5). Then, each alternative was assigned a weight for each criterion, relative to how often the alternative was mentioned in relation to that criterion (Table 6 and S8 File).

**Table 6:** Weighted criteria and overall rank of human observers (‘Obs.’), electronic monitoring (‘EM’), detailed logbooks (‘Log’), and the status quo (‘SQ’) alternatives for data collection at sea across 16 criteria.

Theme	Specific Criteria	Weight	Obs.	EM	SQ	Log
Accuracy	Accurate/Efficient/Reliable Method	0.107	0.246	0.265	0.276	0.213
Change	Change is Accepted	0.044	0.212	0.306	0.224	0.257
Goals	Data Collection Goals are Clear	0.032	0.076	0.357	0.303	0.264
Data	More Data is a Good Thing	0.104	0.212	0.251	0.245	0.291
Use	Data Use is Clear	0.036	0.119	0.236	0.428	0.217
Enforcement	Positive Enforcement/Trust	0.079	0.257	0.280	0.234	0.230
Cost	Lower Cost	0.075	0.173	0.272	0.225	0.330
Intrusiveness	Less Intrusive	0.084	0.147	0.238	0.275	0.340
Liability	Lower Potential for Legal Liability	0.029	0.188	0.320	0.237	0.254
Lifestyle	Fewer Alterations to Lifestyle/Fishing Behavior	0.070	0.153	0.279	0.276	0.292
Logistics	Fewer Logistical/Technical Inconveniences	0.147	0.176	0.247	0.309	0.267
Realistic	Realistic Option	0.016	0.343	0.343	0.101	0.214
Safety	Safe Method	0.022	0.067	0.169	0.382	0.382
Space	Space Saver	0.086	0.158	0.380	0.228	0.234
Time	Minimal Time Commitment	0.025	0.118	0.260	0.473	0.149
Uncertainty	Certainty/Understanding of Method	0.046	0.172	0.172	0.385	0.272
Overall Rank			0.183	0.271	0.279	0.267

Overall rankings were calculated as the sumproduct of the weights of the criteria and alternatives across the suite of criteria and alternatives (Table 6 and S8 and S9 Files). The resulting solution differs from that of a simple pairwise comparison because criteria are allowed to influence the solution. The criterion for how much an alternative affected the logistics (ease or difficulty) of fishing was mentioned by more interviewees than any other criterion (Fig 5 and Table 6 and S9 File). This suggests that whether or not a data collection method was perceived as easy or difficult to implement was the most important factor influencing whether fishermen would support that alternative. Other key criteria included: whether or not fishermen believed that an alternative would produce accurate data; whether or not fishermen felt that more data was a good thing; how much space fishermen thought an alternative would take up; and, whether or not a data collection method was perceived to be an intrusion on fishermen’s privacy (Fig 5 and Table 6 and S8 and S9 Files).

When preference criteria such as data accuracy and ability to positively enforce fishing behavior are allowed to influence the decision problem, the overall rankings get closer to one another across all four alternatives, but the status quo again emerges as the preferred option. Overall, the fishermen interviewed for this project perceived the status quo option as a logistically easy, safe, well-understood method with clear goals, and entailing a comparatively small time commitment. Across the full suite of 16 weighted criteria, interviewees ranked the electronic monitoring alternative a close second (scoring 0.271 as compared to 0.279 for status quo). Relative to other alternatives, interviewees perceived electronic monitoring as a space-saving, realistic option, with a clear set of goals, and a low potential for causing legal liability issues (Table 6 and S8 and S9 Files). In other words, interviewees perceived electronic monitoring to be a change they could accept.

The decision problem solution for this AHP model—an overall rank for each alternative—may change depending on which criteria are maintained for consideration. For example, if a shorter list of criteria is considered across data collection alternatives, the preferred solution from the perspective of halibut fishermen shifts. For example, if accuracy, lifestyle, logistics, and space are the only criteria considered in our model, the preferred data collection alternative shifts from the status quo (scoring 0.278) to electronic monitoring (scoring 0.285) (Table 7).

**Table 7:** Weighted criteria and overall rank of human observers (‘Obs.’), electronic monitoring (‘EM’), detailed logbooks (‘Log’), and the status quo (‘SQ’) alternatives for data collection at sea across four criteria.

Theme	Specific Criteria	Weight	Obs.	EM	SQ	Log
Accuracy	Accurate/Efficient/Reliable Method	0.261	0.246	0.265	0.276	0.213
Lifestyle	Fewer Alterations to Lifestyle/Fishing Behavior	0.171	0.153	0.279	0.276	0.292
Logistics	Fewer Logistical/Technical Inconveniences	0.359	0.176	0.247	0.309	0.267
Space	Space Saver	0.210	0.158	0.380	0.228	0.234
Overall Rank			0.187	0.285	0.278	0.250

Asking fishermen to rank a suite of alternatives thus provides more information than simply asking them to identify their preferred alternative. Rankings and pairwise comparisons elucidate their perceptions of second-best and third-best alternatives. This can be important to decision-makers who may find convergence on a second or third-best alternative as more viable than choosing among disparate first-choice alternatives. Asking fishermen to express their preferences between each pair of alternatives (pairwise comparisons) provided even more information about the strength (weight) and consistency of those preferences. However, even pairwise comparisons did not shed light on the basis for preferences. We followed our presentation of simple rankings and pairwise comparisons across the alternatives with a presentation of weighted decision criteria in an AHP model. The AHP model identified a preferred alternative based on fishermen’s judgments about the importance of decision criteria and the performance of the alternatives in relation to those criteria.

## Conclusion

In this paper, we have demonstrated how a management body might gather reliable descriptions of fishermen’s preferences with a relatively small investment of time and money: analyzing semistructured interviews with a relatively small sample of fishermen in a three-stage AHP process. We collected preference data from halibut fishermen across four communities in a region, distilled that information using inductive content text analysis, and then applied a quantitative decision analytic method—AHP—to identify the strength of preferences across alternatives and the features of the alternatives that underpin fishermen’s preferences. The findings demonstrated remarkably consistent, relatively homogenous preferences fishermen had about data collection methods on their vessels. This entire process can be completed in a relatively short time period (e.g., 12–18 months), which aligns with the timeframe of management decision-making in this fishery.

Fisheries scientists and managers have increasingly adopted stock assessment and management strategies that are robust to the presence of biological uncertainty and environmental variation [55–58]. However, it remains uncommon for fisheries managers to analyze fishermen’s preferences for incorporation in the decision-making process in structured or systematic ways. When the restructured observer program was implemented in the halibut fleet during 2013, it was anticipated that there would be challenges associated with the shift [59]. Application of the methods presented in this paper during the regulatory review process (Fig 3) could have provided managers with greater understanding of fishermen’s preferences and the tools necessary to anticipate and mitigate some major alterations to program design (e.g., resulting from vessel exemptions) that emerged during the first few years of the observer program in the halibut fleet [32,34].

Systematic *ex-ante* analysis of halibut fishermen’s preferences might have provided reliable insights into characteristics of potential data collection alternatives that were desirable, objectionable, or important to them. This knowledge could have facilitated consideration of additional data collection alternatives for halibut vessels. Systematic consideration of those preferences could provide managers with the tools necessary to understand areas where fishermen’s preferences and management objectives converge or diverge. Through assessing type and strength of fishermen’s preferences related to management actions, it becomes possible for fisheries managers to choose confidently from the best (or least worst) decisions from multiple stakeholder perspectives at once. Managers can also use AHP to prioritize management plans [60] or to assess multispecies management and resource allocation among user groups [44]. This could lead to regulatory decisions with high likelihoods of meeting program objectives or data needs with minimal disruption or cost to fishing operations and lifestyles, as well as high levels of acceptance and compliance from the fishing fleet. In the case study of Pacific halibut, AHP could also be a useful tool to highlight ongoing perceptions fishermen have about data collection alternatives and to determine what drives those perceptions. This could inform future modifications of alternatives for data collection in the halibut fleet (e.g., reducing costs).

Understanding the relative importance of multiple objectives in fisheries management decisions supports development of the most appropriate strategies [46]. An AHP decision-making process similar to that presented in this paper could be used regularly and across multiple fisheries to provide insights into characteristics of decision alternatives that are desirable, objectionable, or important for fishermen. Managers could potentially increase acceptance of alternatives by stressing how well they perform in relation to each criterion and how important each criterion is to meeting data/management needs. Managers could also look at where proposed alternatives fare poorly on certain criteria and work to assure that proposed alternatives better reflect fishermen’s preferences and judgments.

AHP can function as a useful decision-support tool without replacing existing decision-making methods [49]. Using a three-stage AHP to document preferences of fishermen is compatible with broader trends in the management and sustainable use of fishery-ecological systems, including Management Strategy Evaluations (MSE) [57,61]. The MSE framework outlines strategies for effectively managing uncertainty in decision-making. Within the MSE framework, there is increased space for systematically considering fishermen’s preferences, and for allowing social outcomes and preferences to play a direct role in decision-making. Characterizing fishermen’s preferences may facilitate decreased uncertainty during the decision-making process while increasing compliance on fishing grounds. AHP modeling is one way to characterize those preferences.

This paper did not aim to be statistically random in its sampling strategy (although the methods we illustrate could be used in conjunction with a statistically random sample frame). Instead, we sought to gain information from willing and interested fishermen. We have measured preferences in terms of consistency and strength across a set of criteria defined by the fishermen who participated in this study. In this instance, these opinions are so strongly invariable that it is likely they represent a large portion of the halibut fishermen in the region. That said, marginalized populations (including those based in ethnicity and gender) in the halibut fishery were not assessed in terms of their preferences, which may differ from the prevailing preferences reported in this paper.

Interview sampling for this project was not stratified or targeted to reach specific ethnic or gender distributions, and there were not enough responses from underrepresented groups to be able to explore subgroup differences in any meaningful way through subsampling the interviews conducted for this paper. However, future studies could contribute to a growing body of literature focusing on marginalized and underrepresented groups [62] to allow for demographic comparisons using the same methods presented here. AHP can provide an avenue for affected fishermen to play a role in designing policies that shape and reshape relationships between society and the life supporting marine ecosystems upon which human well-being depends. The three stage AHP methods described in this paper could be used to improve practices in the management of fisheries and other natural resources around the world.

Note: The term “fishermen” is used to refer to fishermen and women in Alaska throughout this paper. While there is some debate as to how people who commercial fish for a living prefer to be labeled, it is the understanding of the authors that the women who participated in this study refer to themselves as “fishermen.” Media coverage regarding women who fish in Alaska suggests the term “fishermen” or “fisherwomen” could be appropriate titles for women who fish commercially throughout the state (http://www.alaskafishradio.com/fishermen-of-both-sexes-dislike-gender-neutral-title/).

## Supporting information

S1 File Data description for sample pool of halibut IFQ holders in the four study communities in 2015 (n = 503). (RTF)

S2 File Interview protocol. (RTF)

S3 File Summary of interviewee characteristics (n = 76; 75 were used in Ranking and AHP). (Table A) Number of interview participants across categorical variables for fishing characteristics during study year 2015. (Table B) Percent of 2C IFQ shares held by interview participants in 2015, split across share types (A, B, C, and D) and in total. (Table C) Characteristics of interview participants in 2015 across continuous variables for fishing characteristics (n = 76). (Figure A) Halibut quota share holdings by class (A Class = Freezer longliners; B Class = Vessels greater than 18.3 m. long; C Class = Vessels 10.7–18.3 m. long; D Class = Vessels under 10.7 m. long) across (a) all four study communities (n = 804 blocks) and (b) within the interview group of 76 IFQ holders (n = 135 blocks).
(RTF)

S4 File Areas fished by study participants, organized by community of residence (*n* = 76). (RTF)

S5 File Data used to calculate outcomes from pairwise comparison interview exercise. (CSV)

S6 File ANOVA and chi-square test data used to compare vessel length and previous experience with human observers (*n* = 72). (CSV)

S7 File Results of ANOVA and chi-square tests comparing vessel length and previous experience with human observers (n = 72). (Table A). ANOVA results (n = 72) comparing vessel length and previous experience with human observers. If the p-value is less than 0.05, there is sufficient evidence to reject the null hypothesis (that the mean, or average, value of the dependent variable experience with human observers is the same for all vessel lengths). (Table B) Chi-square results (n = 72) comparing vessel length and previous experience with human observers. If the p-value is less than 0.05, there is sufficient evidence to reject the null hypothesis (that the two characteristics are independent).
(RTF)

S8 File Multi-directional criteria (*n* = 33) from interviews with halibut fishermen (*n* = 75). (RTF)

S9 File Excel file demonstrating how criteria and AHP solutions were calculated (*n* = 75). (XLSX)
