# Supplementary material for: Characterizing preferences of fishermen to inform decision-making: A case study of the Pacific halibut (Hippoglossus stenolepis) fishery off Alaska
Source: PLoS One. 2019 Mar 1;14(3):e0212537. doi: 10.1371/journal.pone.0212537 (PMC6396916; doi:10.1371/journal.pone.0212537)
Supplement: S1 File — (RTF) [file pone.0212537.s001.rtf]

S1 Data description for sample pool of halibut IFQ holders in the four study communities in 2015 (n = 503).

These data originated from the NOAA website: https://alaskafisheries.noaa.gov/permits-licenses?field_fishery_pm_value=Individual+Fishing+Quota+%28IFQ%29+Halibut%2FSablefish+and+CDQ+Halibut+IFQ 
These data were downloaded in the file: https://alaskafisheries.noaa.gov/sites/default/files/reports/15ifqunitf.csv 
Data cleaning:
·	All non-halibut quota holders were removed from the list, as well as any individuals without 2C quota or with addresses outside the study communities.
·	Communities "Douglas" and "Auke Bay" were retained, as they are part of the City and Borough of Juneau area. 
·	Repeats in the resulting list were removed (using columns F,G,H, and I) to create a list of individuals holding halibut IFQ shares in the study communities during 2015.
·	The column "QS Units" was deleted, since it was no longer accurate after repeats were removed.
