# Supplementary material for: Characterizing preferences of fishermen to inform decision-making: A case study of the Pacific halibut (Hippoglossus stenolepis) fishery off Alaska
Source: PLoS One. 2019 Mar 1;14(3):e0212537. doi: 10.1371/journal.pone.0212537 (PMC6396916; doi:10.1371/journal.pone.0212537)
Supplement: S2 File — (RTF) [file pone.0212537.s002.rtf]

1	S2 Interview Protocol. 2
3


Please read this form carefully

4	You are being asked to take part in a study about at-sea monitoring in the Pacific halibut fishery.
5	This project has been approved by the XXXXXX. 6
7	The goal of this study is to document your observations and opinions about monitoring. You are
8	being asked to take part in this study because of your fishing experience. You are invited to ask
9	any questions at any time during your participation. The information that we collect might
10	describe how at-sea monitoring affects the halibut fleet. 11
12	If you decide to take part, you will be asked to describe your fishing experience during an
13	interview lasting about one hour. We will make every effort to hold the interview in a way that is
14	comfortable for you. Some questions will include where you fish and what kinds of things you
15	catch other than halibut. Other questions will focus on your opinions about at-sea monitoring.
16	The interview may be recorded to help in taking notes. You may ask for the voice recorder to be
17	turned off at any time. 18
19	We do not expect any risks to you if you take part in this study. At the same time, you may not
20	get any benefits from taking part in this study. Information we get about you from the research
21	will be kept confidential, and stored in a locked office. Information with your name attached will
22	not be shared with anyone outside the research team. We will code your information with a
23	number so no one can trace your answers to your name. Your name will not be used in reports,
24	presentations, and publications. 25
26	Your decision to take part in the study is voluntary. If you decide to take part you can stop at any
27	time. You may change your mind and ask to be removed from the study. You may also skip any
28	questions. If you have questions now, feel free to ask. If you have questions, you may email
29	XXXXXX.
30
31	STATEMENT OF CONSENT: I understand everything described above. My questions have
32	been answered to my satisfaction, and I agree to participate in this study. I am 18 years old or
33	older. I have been provided a copy of this form. 34
35	 	Signature 36
37
38	 	Printed Name
38	
39	Everything in this interview refers to the commercial fishery for Pacific halibut in Alaska. This
40	means that even if you have experience fishing for halibut in a non-commercial way, I would
41	like you to try to focus your answers on your commercial experiences. The interview is set up in
42	two main parts. First, I will ask about your fishing experiences. Then I will ask about the
43	observer program and data collection at sea more generally. 44
45	Part 1: Fishing Experience

46	1. What year did you start commercial fishing?

47	2. What led you into commercial fishing [family tradition, first generation, etc.]?

48	3. What year did you start commercially fishing for halibut?

49	4. Has the boat you use changed over time? How?

50	5. What type of boat do you currently use to fish for halibut?

Type (e.g., schooner)	Length	Width	Engine Power	GT (Hold Capacity)	Number of bunks	
						
51

52	6. Which regulatory areas do you currently hold quota in?

53	7. Have you ever held quota in another area?

54	8. When you think about your fishing experience, which area do you think of most of it as

55	taking place?

56	9. What proportion of your halibut fishing takes place in Area 2C?

57	10. Which months do you currently fish for halibut commercially?

Jan	Feb	Mar	Apr	May	Jun	Jul	Aug	Sep	Oct	Nov	Dec	
												
58
59	11. How has this changed over time? 60
61	12. Please describe your typical gear setup 62
Snap-on?	Hook spacing	Hook type/size	Avg. # of skates	Length of avg. skate	Bait	
						

63
64	13. How has this changed over time?

65	14. Do you combo fish black cod [sablefish] and halibut?

66	Part 2: Understanding monitoring experiences and preferences
67
68	Thank you. Now I would like to focus on the observer program. 69
70	1. Did you participate in an observer program in a different fleet besides halibut before

71	2013?

72	2.   What is your understanding of the purpose of the observer program [and how it works]?

73	3.   What are some positive outcomes of the observer program? What are some negative

74	outcomes of the observer program?

75	4. Have you been chosen to host an observer since 2013, during halibut fishing trip(s)?

76	5. If so, please describe the experience.

77	6. Federal fisheries managers have voiced an interest in collecting biological data at sea in

78	the halibut fishery. How would you feel about a system where you were asked to bring

79	samples into port for scientists on land?

80	7. How has the halibut observer program affected your outlook on the future?

81
82	For this section, I would like you to imagine that all of the types of monitoring described below
83	could apply to you. Even if none of them have ever applied to you, please pretend that they all
84	could during this exercise.
85	This page is just for you to read. You do not need to write on it.
86
87	1. Human Observers: currently in place, using only human observers to document all of your
88	fishing practices at-sea 89
90	2. Electronic Monitoring: instead of humans, cameras would be installed on your vessel to
91	record all of your fishing practices at-sea 92
93	3. Detailed Logbooks: the fisherman is responsible for recording everything they catch (not just
94	halibut) in a logbook 95
93	
96	4. Before 2013: the way things were; halibut are reported and port sampled but no other species
97	are recorded

98
99	For the next exercise, I would like you to think about the strengths and weaknesses of these four
100	different monitoring alternatives. Using the prompts on the next two pages, please rate the
101	different types of at-sea monitoring: EM, human observer, detailed logbooks, same as before 102	2013.
103
104	Please circle the number that shows how much you support each type of monitoring.
105
1.	Human Observers

1	2	3	4	5	6	7	
No Support	Some Support	Strong Support


2.	Electronic Monitoring

1	2	3	4	5	6	7	
No Support	Some Support	Strong Support


3.	Detailed Logbooks

1	2	3	4	5	6	7	
No Support	Some Support	Strong Support


4.	Before 2013

1	2	3	4	5	6	7	

106
107
108
No Support	Some Support	Strong Support

109
110


111
112
113
114
115
116
Please circle number that shows your relative preferences between each pair of options

1. Human Observers	Electronic Monitoring

1	2	3	4	5	6	7	
Strongly Preferred


2. Human Observers	No Difference	Strongly  Preferred


Detailed Logbooks	
1	2	3	4	5	6	7	
Strongly Preferred


3. Human Observers	No Difference	Strongly  Preferred


Before 2013	
1	2	3	4	5	6	7	
Strongly Preferred


4. Electronic Monitoring	No Difference	Strongly  Preferred


Detailed Logbooks	
1	2	3	4	5	6	7	
Strongly Preferred


5. Electronic Monitoring	No Difference	Strongly  Preferred


Before 2013	
1	2	3	4	5	6	7	
Strongly Preferred


6. Detailed Logbooks	No Difference	Strongly  Preferred


Before 2013	
1	2	3	4	5	6	7	
Strongly Preferred	No Difference	Strongly Preferred	

Please tell me a bit about why you ranked the way that you did.

Part 3: Demographic Information
1. What year were you born?  	


117	2. How did you obtain your IFQ?  	

118	3.   Have you personally fished your 2C IFQ every year since obtaining it?	YES	NO
117	
119	4. How long do you plan to keep fishing your IFQ?  	

120	5.   Do you identify with an Alaska Native tribe?	YES	NO

121	6. How many people live in your household?  	

122	7. How satisfied are you with your financial situation?
123	a) Very Unsatisfied
124	b) Unsatisfied
125	c) Satisfied
126	d) Very Satisfied

127	8. What percent of your household income comes from fishing (household income includes
128	your income as well as income from anyone else who lives in your household)? 129		a) 1-24%
130	b)  25-50%
131	c)  51-74%
132	d)  75-99%
133	e) 100%

134	9.   Do you own the fishing vessel that you fish halibut on?	YES	NO

135	10. Are you a member of a fisheries organization? If so, which one?	YES	NO

136	 	

137	11. Are you a full time resident in 	?	YES	NO

138	12. Is 	currently dependent on commercial fishing?	YES	NO

139	13. Did you grow up in 	?	YES	NO

140	14. Did one of your parents grow up in 	?	YES	NO

141	15. Did one of your grandparents grow up in 	?	YES	NO

142	May I contact you in the future with results and/or more questions?	YES	NO 143
144	If so, please provide contact Information: Email, Phone number, Address: 145
146	Part 5: Closing

147	5.1 Do you have any questions for me?


148	

149	
Thank you for your time ☺
