# Supplementary material for: Characterizing preferences of fishermen to inform decision-making: A case study of the Pacific halibut (Hippoglossus stenolepis) fishery off Alaska
Source: PLoS One. 2019 Mar 1;14(3):e0212537. doi: 10.1371/journal.pone.0212537 (PMC6396916; doi:10.1371/journal.pone.0212537)
Supplement: S3 File — (Table A) Number of interview participants across categorical variables for fishing characteristics during study year 2015. (Table B) Percent of 2C IFQ shares held by interview participants in 2015, split across share types (A, B, C, and D) and in total. (Table C) Characteristics of interview participants in 2015 across continuous variables for fishing characteristics (n = 76). (Figure A) Halibut quota share holdings by class (A Class = Freezer longliners; B Class = Vessels greater than 18.3 m. long; C Class = Vessels 10.7–18.3 m. long; D Class = Vessels under 10.7 m. long) across (a) all four study communities (n = 804 blocks) and (b) within the interview group of 76 IFQ holders (n = 135 blocks). (RTF) [file pone.0212537.s003.rtf]

S3 Summary of interviewee characteristics (n = 76; 75 were used in Ranking and AHP).

Table A. Number of interview participants across categorical variables for fishing characteristics during study year 2015. Fishermen were allowed to report fishing during multiple categories for some characteristics (e.g., fishing season), so the numbers do not all add to 76.
Characteristic	Interview participants	Number individual 2C IFQ holders	Percent individual 2C IFQ holders Refer to S1; a list of individual halibut IFQ holders in the study communities was obtained from the NOAA record, at: https://alaskafisheries.noaa.gov/sites/default/files/reports/15ifqunitf.csv) 	
				
Community				
Juneau	12	112	11%	
Hoonah	10	19	53% This number is higher in terms of active fishermen, because some of the listed IFQ holders for this community in 2015 were deceased and not yet removed from the list.	
Petersburg	27	179	14%	
Sitka
Total	27
76	194
503	15%
15%	
Fishing season				
Spring fishing	53	Unknown	Unknown	
Summer fishing	32	Unknown	Unknown	
Fall fishing	36	Unknown	Unknown	
Gear type				
Conventional gear	31	Unknown	Unknown	
Snap-on gear	39	Unknown	Unknown	
Both gear types	6	Unknown	Unknown	
Combo fishing (for sablefish/halibut)	36	Unknown	Unknown	

Table B. Percent of 2C IFQ shares held by interview participants in 2015, split across share types (A, B, C, and D) and in total.
Share type	Total shares in study communities	Percent shares held by interview participants	
			
A (Freezer longliners)	476324	28%	
B (Vessels > 18.3 m)	1427204	19%	
C (Vessels 10.7 – 8.3 m)	25014654	20%	
D (Vessels < 10.7 m)	5082536	22%	
Total	32000718	20%	

Table C. Characteristics of interview participants in 2015 across continuous variables for fishing characteristics (n = 76).
Characteristic		Min.	Max.	Mean	Median	
						
Vessel length (m.)		4.9	26.2	13.4	12.8	
Age		27	81	56	59	
Years of experience		4	57	34	35	


		
 	(a)	(b)
Figure A. Halibut quota share holdings by class (A Class = Freezer longliners; B Class = Vessels greater than 18.3 m. long; C Class = Vessels 10.7-18.3 m. long; D Class = Vessels under 10.7 m. long) across (a) all four study communities (n = 804 blocks) and (b) within the interview group of 76 IFQ holders (n = 135 blocks).
