# Supplementary material for: Characterizing preferences of fishermen to inform decision-making: A case study of the Pacific halibut (Hippoglossus stenolepis) fishery off Alaska
Source: PLoS One. 2019 Mar 1;14(3):e0212537. doi: 10.1371/journal.pone.0212537 (PMC6396916; doi:10.1371/journal.pone.0212537)
Supplement: S4 File — (RTF) [file pone.0212537.s004.rtf]

1	S4. Areas fished by study participants, organized by community of residence (n = 76). 2
3	S4 Fig a. Areas fished by study participants from Hoonah, Juneau, Sitka, and Petersburg.


4
5	Hoonah	Juneau


6
7	Sitka	Petersburg


1
