# Supplementary material for: Characterizing preferences of fishermen to inform decision-making: A case study of the Pacific halibut (Hippoglossus stenolepis) fishery off Alaska
Source: PLoS One. 2019 Mar 1;14(3):e0212537. doi: 10.1371/journal.pone.0212537 (PMC6396916; doi:10.1371/journal.pone.0212537)
Supplement: S7 File — (Table A). ANOVA results (n = 72) comparing vessel length and previous experience with human observers. If the p-value is less than 0.05, there is sufficient evidence to reject the null hypothesis (that the mean, or average, value of the dependent variable experience with human observers is the same for all vessel lengths). (Table B) Chi-square results (n = 72) comparing vessel length and previous experience with human observers. If the p-value is less than 0.05, there is sufficient evidence to reject the null hypothesis (that the two characteristics are independent). (RTF) [file pone.0212537.s007.rtf]

S7 ANOVA and chi-square tests comparing vessel length and previous experience with human observers (n = 72).

Table A. ANOVA results (n = 72) comparing vessel length and previous experience with human observers. If the p-value is less than 0.05, there is sufficient evidence to reject the null hypothesis (that the mean, or average, value of the dependent variable experience with human observers is the same for all vessel lengths).
ANOVA	Df	Sum Sq	Mean Sq	F value	Pr(>F)	
Experience with observers	1	0	0.27	0.002	0.967	
Residuals	72	10978	152.47			

Table B. Chi-square results (n = 72) comparing vessel length and previous experience with human observers. If the p-value is less than 0.05, there is sufficient evidence to reject the null hypothesis (that the two characteristics are independent).
Chi-square	X-squared	Df	P-value	
	0	1	1	
