# Supplementary material for: Characterizing preferences of fishermen to inform decision-making: A case study of the Pacific halibut (Hippoglossus stenolepis) fishery off Alaska
Source: PLoS One. 2019 Mar 1;14(3):e0212537. doi: 10.1371/journal.pone.0212537 (PMC6396916; doi:10.1371/journal.pone.0212537)
Supplement: S8 File — (RTF) [file pone.0212537.s008.rtf]

S8 Multi-directional criteria (n = 33) from interviews with halibut fishermen (n = 75).

S8 Table a. 33 criteria are presented along with how many interviewees mentioned each one. 
Code Criteria	Obs.	EM	SQ	Log	Total	
Accuracy_Efficiency_Reliability_	2	4	2	3	11	
Accuracy_Efficiency_Reliability_-	22	11	0	12	45	
Accuracy_Efficiency_Reliability_+	19	17	3	9	48	
Change_Accepted	13	6	3	3	25	
Change_Not Accepted	9	1	2	1	13	
Data_Collection Goals Unclear	22	1	2	3	28	
Data_More Good	21	8	14	14	57	
Data_More Not Necessary	18	4	8	4	34	
Data_Use Unclear	17	6	1	7	31	
Enforcement_Trust	12	2	2	2	18	
Enforcement_Trust_-	25	11	4	7	47	
Enforcement_Trust_+	12	7	1	2	22	
Financial_	10	10	1	0	21	
Financial_-	40	11	0	1	52	
Financial_+	5	5	0	3	13	
Intrusiveness_Privacy_	7	1	0	0	8	
Intrusiveness_Privacy_-	32	17	0	2	51	
Intrusiveness_Privacy_+	4	9	2	7	22	
Liability_-	11	3	0	1	15	
Liability_+	4	5	0	1	10	
Lifestyle_Altered Behavior_-	42	5	1	0	48	
Lifestyle_Altered Behavior_+	5	4	2	2	13	
Logistics_Technical_Inconvenience_Difficult	40	17	0	28	85	
Logistics_Technical_Inconvenience_Easy	8	10	5	20	43	
Politics_Inevitable	9	1	1	1	12	
Politics_Unrealistic	1	1	9	3	14	
Safety_	2	1	0	1	4	
Safety_-	15	4	0	0	19	
Space_	17	5	1	0	23	
Space_-	49	7	0	3	59	
Space_+	5	10	0	1	16	
Time Commitment	11	3	0	8	22	
Uncertainty	15	15	3	7	40	


S8 Table b. Total number of occurrences of criteria for each data collection alternative.
Code Name	Total Number of Occurrences	
Observer Program Criteria	466	
Electronic Monitoring Criteria	169	
Status Quo Criteria	53	
Logbook Criteria	133	
